# Frontotemporal lobar degeneration and amyotrophic lateral sclerosis: A bibliometric analysis

**DOI:** 10.1097/MD.0000000000043180

**Published:** 2025-07-04

**Authors:** Gaihong Liu, Ningning Liu, Yingxue Xu, Fan Su

**Affiliations:** a The First Clinical Medical College, Shandong University of Traditional Chinese Medicine, Jinan, China; b Department of Anesthesiology, Affiliated Hospital of Shandong University of Traditional Chinese Medicine, Jinan, Shandong Province, China.

**Keywords:** amyotrophic lateral sclerosis, bibliometric analysis, frontotemporal lobar degeneration, future research direction, research hotspots

## Abstract

**Objective:**

This study analyzes the research hotspots and future directions of frontotemporal lobar degeneration (FTLD) and amyotrophic lateral sclerosis.

**Methods:**

Relevant literature was searched using the Web of Science database and analyzed using econometric tools such as CiteSpace and VOSviewer.

**Results:**

A total of 145 articles were included in this study, involving 317 research institutions in 31 countries and regions. Acta Neuropathologica is a prominent journal in terms of issuance and influence, and countries such as the United States and Japan, as well as institutions such as the University of Pennsylvania, occupy an important position in the research. The keywords cover various aspects such as disease characteristics and gene mutations; highly-cited literature focuses on TDP-43 protein and C9orf72 gene mutations. Research hotspots include TDP-43 protein disease-driven pathomechanisms, RNA-related studies, clinical manifestations of the disease and genetic studies, etc. In recent years, research focus has shifted to RNA, C9orf72 gene and so on.

**Conclusion:**

To our knowledge, this study is the first econometric evaluation of the FTLD-ALS literature, and although there are limitations such as relying on the number of documents and citation relationships, and a single source of data, it provides a valuable reference for research in this field and helps to promote subsequent research.

## 1. Introduction

Frontotemporal lobar degeneration (FTLD) and amyotrophic lateral sclerosis (ALS) are 2 serious neurodegenerative disorders with partially similar pathophysiologic mechanisms but also many differences.^[1,2]^ FTLD is a category of dementia syndromes whose main pathological feature is selective frontal and/or temporal lobe atrophy.^[3]^ Its main features include progressive mental behavioral abnormalities, executive dysfunction, and impaired speech.^[4–6]^ The pathogenesis of FTLD involves factors such as abnormal protein aggregation, neuronal damage and cell death.^[7]^ ALS causes progressive damage to motor neurons in the cerebral cortex, motor nuclei in the brainstem, and anterior horn cells in the spinal cord, resulting in muscle weakness, muscle atrophy, and progressive movement disorders.^[8,9]^ ALS often begins with muscle weakness and atrophy, eventually affecting respiratory muscles and other important body functions. It is typically characterized by degeneration of motor neurons and protein aggregation around motor neurons.^[10]^

Although FTLD and ALS have their own characteristics in clinical manifestations and pathogenic mechanisms, academic research has shown that there are some convergences between the 2. Clinically, it has also been found that some individuals may simultaneously exhibit the clinical and pathological features of both FTLD and ALS, which has led to the birth of the concept of the FTD-ALS spectrum. In-depth understanding and exploration of the association between these 2 diseases are playing an increasingly crucial role in the academic community. With the continuous advancement of FTLD-ALS-related research, a large number of achievements have been made in this field. However, currently, there are some problems such as the scattered nature of the achievements, which makes it difficult to effectively integrate them, the insufficient prominence of research focuses, and the lack of systematic collation. Bibliometric analysis is an effective method for quantifying, summarizing, and examining the literature in this field. In this study, we utilized bibliometric software such as CiteSpace, VOSviewer version, and Scimago Graphica to sort out and analyze a large amount of literature in the FTLD-ALS field. The aim is to comprehensively gain insights into the collaboration network, evolution process, research hotspots, and future research directions of FTLD-ALS research, so as to provide more accurate and comprehensive guidance for academic exploration in this field.^[11,12]^

## 2. Methods

### 2.1. Data sources and search strategies

**Figure 1.**
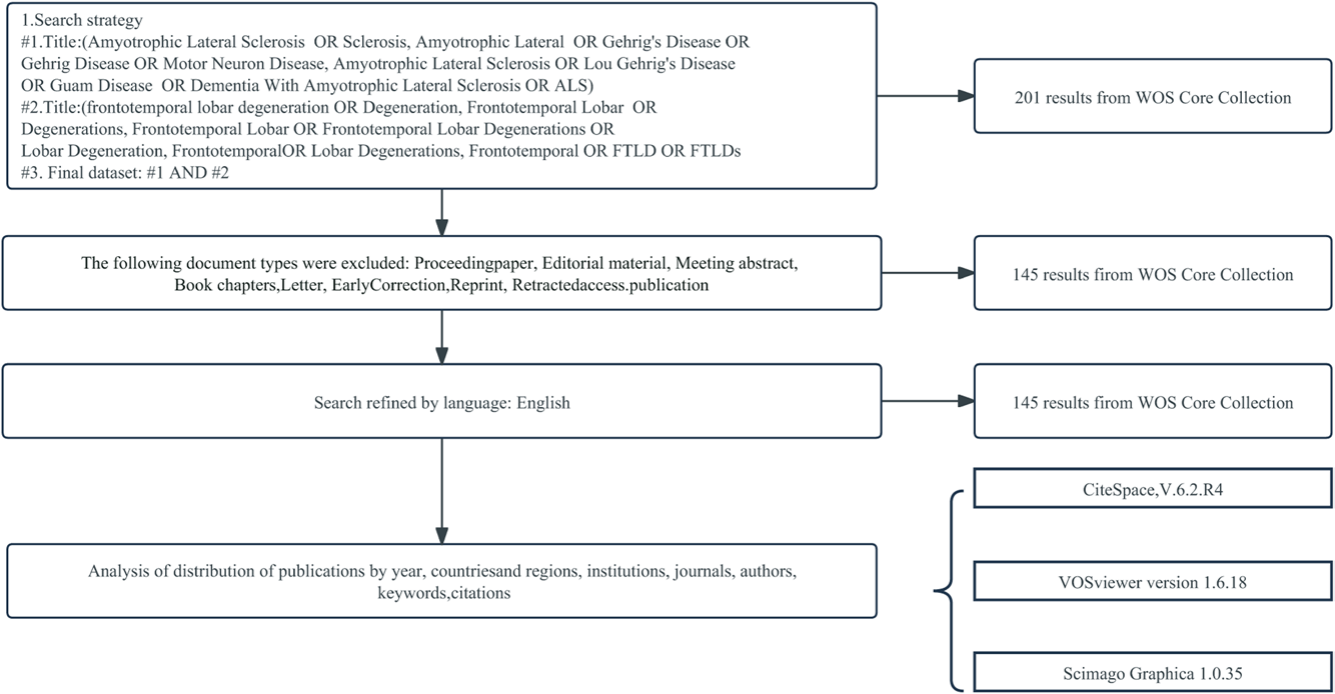
FTLD-ALS literature search flow chart. ALS = amyotrophic lateral sclerosis, FTLD = frontotemporal lobar degeneration.

Web of Science is an international authoritative and comprehensive academic information resource, and the database is regarded as an important search tool for scientific analysis and evaluation.^[13]^ Since this study focuses on the FTLD-ALS field, it is necessary to comprehensively and accurately obtain relevant high-quality academic literature. The rich literature collection and powerful retrieval function of Web of Science can precisely meet this need. The framework of the literature retrieval is as follows: (TI = [frontotemporal lobar degeneration] OR TI = [degeneration, frontotemporal lobar] OR TI = [FTLD] OR TI = [degenerations, frontotemporal lobar] OR TI = [frontotemporal lobar degenerations] OR TI = [lobar degeneration, frontotemporal] OR TI = [lobar degenerations, frontotemporal]) AND (TI = [ALS] OR TI = [sclerosis, amyotrophic lateral] OR TI = [Gehrig disease] OR TI = [Gehrig disease] OR TI = [motor neuron disease, ALS] OR TI = [Lou Gehrig disease OR Guam disease] OR TI = [ALS] OR TI = [dementia with ALS]). The retrieval time ranges from the establishment of the database to October 9, 2023. The Document Types are restricted to Article and Review Article. The language of the literature is restricted to English. LGH and LNN respectively exported 29 items such as the author(s), title, and keywords of the included literature in the formats of “plain text file,” “RIS (other reference software),” and “Excel.” They also manually screened the titles and abstracts, excluded duplicate literatures and those irrelevant to FTLD-ALS research. XYX reevaluated the quality of the literature. If any disagreements occur during the inclusion process, SF will assist in resolving the issue. Finally, 145 relevant studies were included (Fig. 1). LGH used CiteSpace, VOSviewer, and Scimago Graphica software to obtain the potential knowledge information in the FTLD-ALS literature as data materials, and converted the data into a panoramic image. Mainly based on bibliometric indicators and knowledge graph indicators, the research framework and development context of FTLD-ALS were presented in the form of a knowledge graph, and then the research hotspots and future research directions in the FTLD-ALS field were analyzed.

### 2.2. Visualization and network mapping

CiteSpace, V.6.1.R6, VOSviewer version 1.6.18 and Scimago Graphica 1.0.35 software were used for the bibliometric analysis. VOSviewer is a software that supports multi-format data import, co-occurrence analysis, clustering analysis, overlay visualization, and data statistics, and is able to intuitively VOSviewer is a bibliometric analysis and visualization software that supports multi-format data import, co-occurrence analysis, clustering analysis, overlay visualization and data statistics, and can intuitively display the core authors, journals, institutions and countries in the field of FTLD-ALS, such as the partnership, distribution and research hotspot association.^[14,15]^ CiteSpace software is able to analyze and display the potential knowledge embedded in scientific literature, while its time-series analysis function can show the changes of keyword co-occurrence and clustering over time, helping researchers to understand the evolutionary trend of research hotspots.^[16,17]^ SCImago Graphica is a graphing software for lightweight applications. This study adopts a synergistic and complementary strategy to help researchers grasp the overall research situation in the field of FTLD-ALS at a glance, explore potential research gaps, and provide solid data support and directional guidance for subsequent research planning.

## 3. Results

### 3.1. Trend analysis

**Figure 2.**
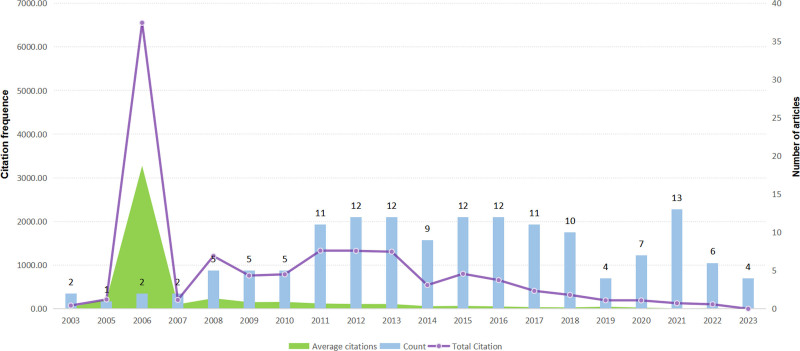
FTLD-ALS trends map. ALS = amyotrophic lateral sclerosis, FTLD = frontotemporal lobar degeneration.

A total of 145 FTLD-ALS literatures were included in this study. More than two-thirds of these literatures are original articles, which fully demonstrates that the research vitality in this field mainly stems from innovative first-hand explorations. By analyzing the literatures according to the year, we can gain insights into the changes in research popularity and influence (Fig. 2). From 2003 to 2006, the number of published articles was small, but the citation frequency per article was high. For example, in 2006, the average citation frequency of 2 articles was 3276 times, which might indicate breakthrough achievements. Therefore, we analyzed the articles published in 2006. Both studies found that ubiquitin-positive and tau-negative neuronal cytoplasmic inclusions, etc, are common pathological features of FTLD-ALS, and determined that TDP-43 is an important related protein and there are abnormal modification changes such as phosphorylation, supporting the view that these 2 neurodegenerative diseases have an internal connection and can be classified into 1 category.^[18,19]^ From 2008 to 2013, the number of published articles was stable at 5 to 12, with an average citation frequency of 54 to 241 times, and the field developed steadily. However, after 2014, the average citation frequency declined. In 2021, the average citation frequency of 13 articles was only 9.85 times, and 4 articles published in 2023 have not been cited yet. Although the number of published articles has fluctuated in recent years and reached 13 in 2021, the citation situation is not satisfactory. This may be due to the refinement of research directions, the new achievements not being widely recognized, or the new research still being in the accumulation and precipitation stage, failing to arouse a great response in the academic community.

### 3.2. Analysis of cooperation networks

**Figure 3.**
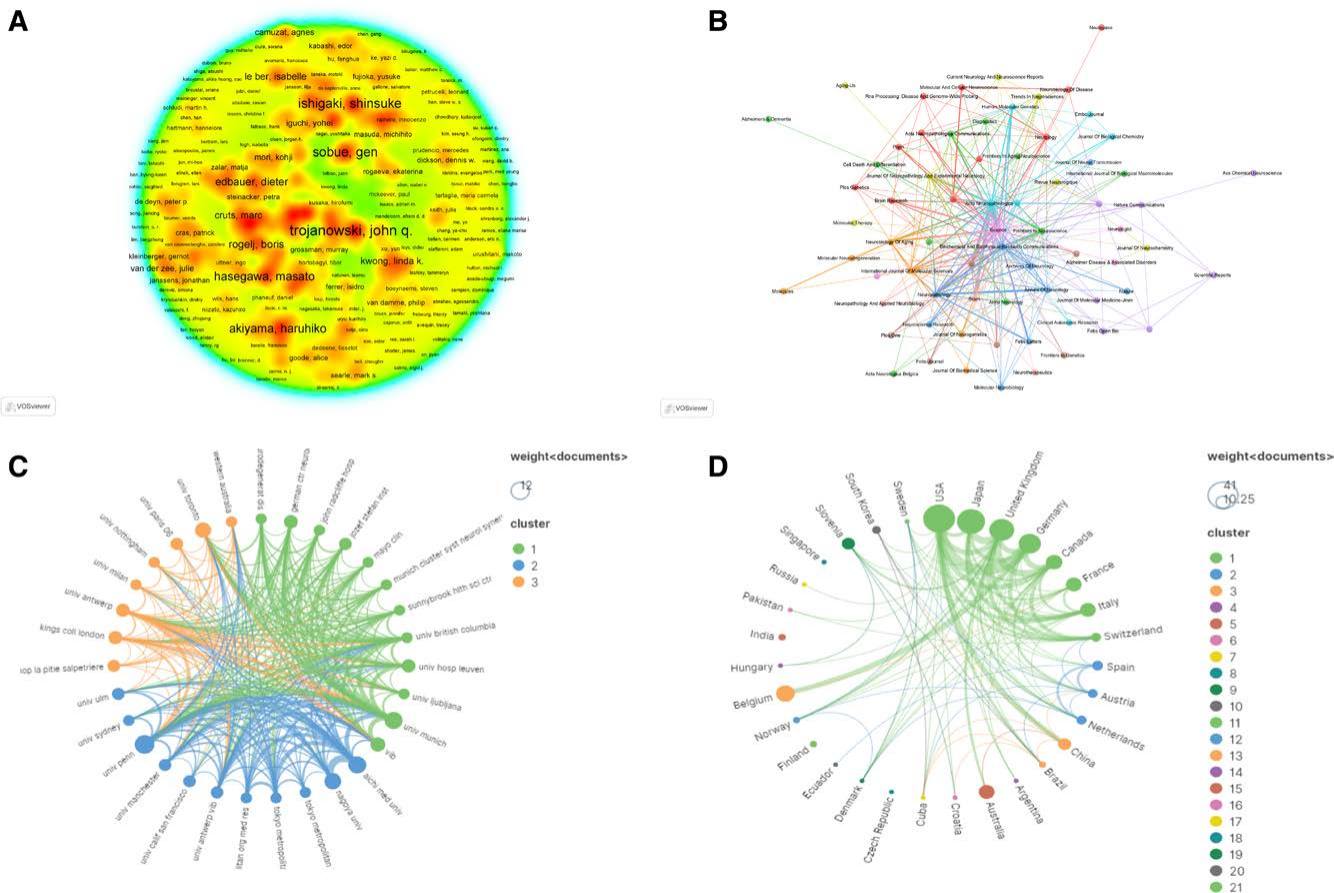
FTLD-ALS collaboration network mapping. (A) Core authors. (B) Journal collaboration networks. (C) Institutional networks. (D) National collaboration networks. ALS = amyotrophic lateral sclerosis, FTLD = frontotemporal lobar degeneration.

In order to provide precise insights into the scientific activity and collaboration patterns in the field of FTLD-ALS on a global scale, we conducted an exhaustive analysis of authors, journals, institutions, and countries in the screened literature. It was found that a total of 897 authors were involved in the relevant literature, with 38 authors with ≥ 4 publications. Trojanowski, Ishigaki, Shinsuke, Neumann, Manuela, Sobue, Gen, Van Broeckhoven, Christine and Yoshida, Mari are the core authors in the field (Fig. 3A). Trojanowski, has the largest number of publications, and his research focus is concentrated on unraveling the pathomechanisms of FTLD-ALS, in particular the aberrant aggregation and ubiquitination modification of the TDP-43 protein and its role in neurodegenerative diseases.^[20–27]^ These studies provide in-depth insights into the pathogenesis and pathological features of FTLD-ALS, and offer potential targets and clues for future diagnosis and treatment. The research achievements in the FTLD-ALS field are mainly published in 76 journals (Fig. 3B). Acta neuropathologica is the journal with the largest number of published articles (N = 16), with a total of 1526 citations and an average of 95.38 citations per article, indicating that acta neuropathologica has wide popularity and academic influence in this field. Secondly, Human Molecular Genetics is also one of the important journals, which has published 5 articles related to FTLD-ALS, with a total of 306 citations and an average of 61.20 citations per article. In addition, journals such as neurobiology of aging, neuropathology, and acta neuropathologica communications have also published research articles related to FTLD-ALS. Although the number of articles published in each journal is relatively small, they still have certain academic influence in the relevant fields. Researchers can choose appropriate academic channels for publishing their research achievements according to the professionalism and influence of different journals. Through the analysis of VOSviewer, there are mainly 317 institutions participating in FTLD-ALS research. Institutions with a frequency of ≥ 4 times were included in the collaborative network analysis (Fig. 3C). In terms of the total number of published articles, the University of Pennsylvania has the largest number of publications (N = 12), followed by Aichi Medical University (N = 10) and Ludwig Maximilian University of Munich (N = 10). Ludwig Maximilian University of Munich has the highest number of citation frequencies (N = 6511), followed by the University of Pennsylvania (N = 5766) and the University of British Columbia (N = 4810). To better understand the distribution of global research forces in FTLD-ALS, we analyzed the countries that published articles. A total of 31 countries have participated in the research in this field (Fig. 3D). The USA has the largest number of publications (N = 41), followed by Japan (N = 32), the United Kingdom (N = 25), and Germany (N = 20). According to the citation analysis, the USA has 8623 citations, followed by Germany (N = 6953) and Canada (N = 5377). In conclusion, the research in the FTLD-ALS field is highly active globally, with a wide and close network of scientific research cooperation. Core authors, leading journals, top institutions, and major scientific research countries all play key roles, laying a solid foundation for the future development of this field. It also provides important references for further deepening international cooperation and collaborative innovation.

### 3.3. Keyword analysis

**Figure 4.**
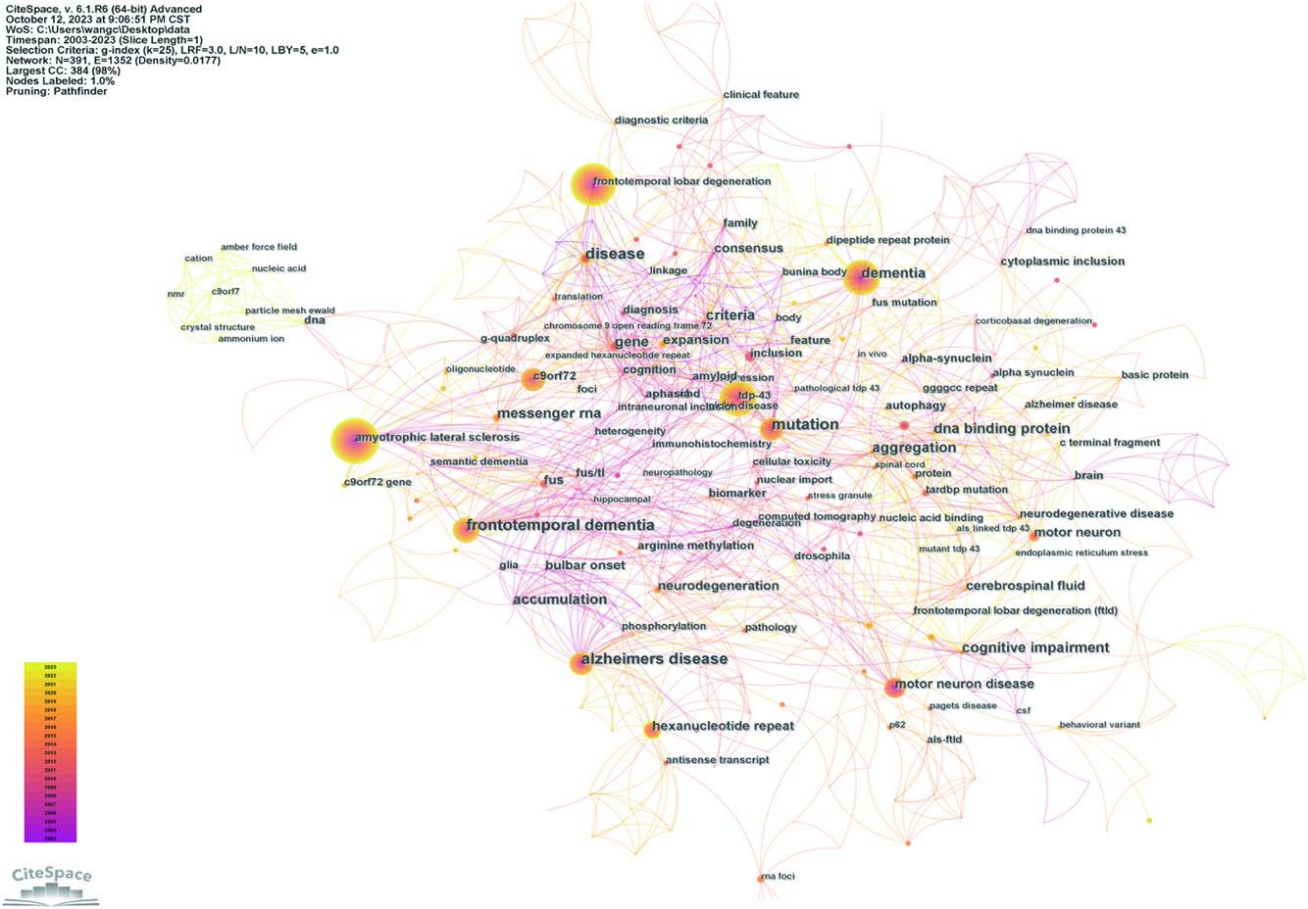
FTLD-ALS keyword co-occurrence map. ALS = amyotrophic lateral sclerosis, FTLD = frontotemporal lobar degeneration.

A total of 391 keywords were extracted from the included literatures (Fig. 4). The most common terms are “ALS” (N = 124) and “frontotemporal lobar degeneration” (N = 104). “tdp-43” (N = 54) is a protein that is closely related to ALS-FTLD, and its abnormal aggregation is associated with the degeneration and pathological changes of nerve cells. “dementia” (N = 47) is a complex symptom of cognitive impairment that often appears in diseases such as FTLD and Alzheimer disease. “mutation” (N = 34) refers to gene mutation, which is related to the pathogenesis and genetics of ALS and FTLD. “frontotemporal dementia” (N = 30) is a subtype of FTLD, usually manifested as cognitive and behavioral problems related to the prefrontal and temporal lobes. “c9orf72” (N = 27) is a gene associated with ALS and FTLD, and its mutation is closely linked to the occurrence and progression of these diseases. In addition, “alzheimer disease” (N = 25) is a neurodegenerative disease that causes a gradual loss of memory and cognitive abilities, and “motor neuron disease” (N = 25) is a group of diseases that affect motor neurons, including ALS. “hexanucleotide repeat” (N = 25) refers to the hexanucleotide repeat sequence, which is related to the genetics of certain diseases. These keywords cover important concepts related to ALS-FTLD, including aspects such as disease characteristics, gene mutations, abnormal protein aggregation, genetic factors, neurodegenerative processes, and cognitive impairment.

**Figure 5.**
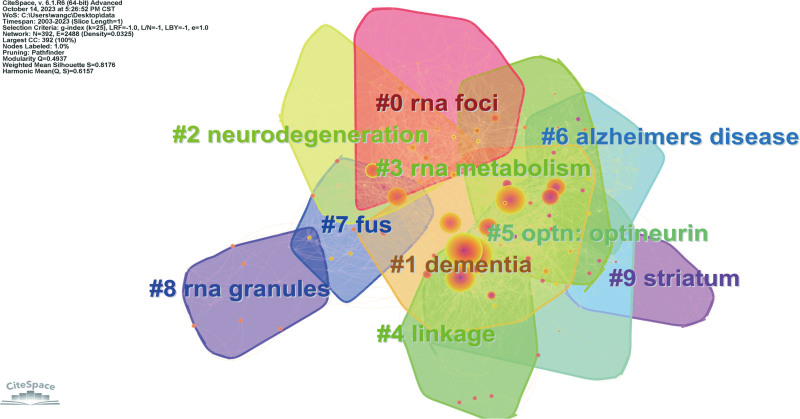
FTLD-ALS keywords clustering map. ALS = amyotrophic lateral sclerosis, FTLD = frontotemporal lobar degeneration.

Keyword clustering analysis provides a comprehensive research framework and helps scholars determine research focuses and the distribution of research contents (Table 1, Fig. 5). Through further analysis of keyword clustering, we found that the main research directions are as follows: #0, #3, and #8 are mainly related to RNA: including “RNA foci,” “RNA metabolism,” and “RNA granules.” These keywords involve the important role of RNA in neurological diseases such as ALS and FTLD and have received extensive attention in the research of ALS and FTD in recent years. Studies have shown that abnormal RNA metabolism may be one of the pathogenic mechanisms of these diseases. #1, #2, and #6 are closely related to the clinical manifestations of ALS and FTLD: including “dementia,” “neurodegeneration,” and “Alzheimer disease.” #4, #5, and #7 mainly focus on the genetic research of neurological diseases such as ALS and FTLD, including “dementia,” “optn: optineurin,” and “fus.”

**Table 1:** Clusters of Co-occurring Keywords in ALS and FTLD research.

Cluster	Size	Silhouette	Mean (year)	Cluster name	Label (LLR)
0	57	0.729	2016	rna foci	rna foci (9.19, 0.005); g-quadruplex (9.19, 0.005); als/ftld (9.19, 0.005); nucleocytoplasmic transport (5.58, 0.05); dipeptide-repeat proteins (5.58, 0.05)
1	55	0.852	2009	dementia	dementia (7.74, 0.01); complex (5.17, 0.05); amyloid (5.17, 0.05); ftld-tdp (5.17, 0.05); neurodegeneration (5.13, 0.05)
2	53	0.82	2016	neurodegeneration	neurodegeneration (19.81, 1.0E−4); frontotemporal dementia (4.95, 0.05); tdp-43 mutation (4.68, 0.05); frontotemporal lobar degeneration ftld) (4.68, 0.05); c9orf72repeat expansion (4.68, 0.05)
3	50	0.748	2014	rna metabolism	rna metabolism (8.94, 0.005); aggregation (6.94, 0.01); c9orf72 (6.02, 0.05); inclusion (5.59, 0.05); motor neuron (5.2, 0.05)
4	43	0.837	2009	linkage	dementia (8.5, 0.005); linkage (7.71, 0.01); nuclear factor tdp-43 (7.71, 0.01); family (7.71, 0.01); gene (7.4, 0.01)
5	41	0.8	2015	optn: optineurin	optn: optineurin (5.06, 0.05); cortical micro-bleeds (5.06, 0.05); transcriptomics (5.06, 0.05); tdp-43 subtypes (5.06, 0.05); white matter changes (5.06, 0.05)
6	36	0.883	2007	alzheimers disease	alzheimers disease (8.76, 0.005); tau protein (8.66, 0.005); motor neuron disease (6.1, 0.05); phosphorylation (5.09, 0.05); transgenic mice (4.32, 0.05)
7	26	0.834	2014	fus	fus (14.51, 0.001); rna (12.97, 0.001); ews (9.23, 0.005); taf15 (9.23, 0.005); syngap (6.46, 0.05)
8	16	0.956	2016	rna granules	rna granules (9.2, 0.005); prion-like domain (9.2, 0.005); sqstm1/p62 (9.2, 0.005); oxidative stress (9.2, 0.005); keap1-nrf2 (9.2, 0.005)
9	15	0.915	2013	striatum	striatum (15.63, 1.0E−4); autopsy (15.63, 1.0E−4); social behavior (7.76, 0.01); art (7.76, 0.01); vglut-1 (7.76, 0.01)

ALS = amyotrophic lateral sclerosis, FTLD = frontotemporal lobar degeneration, LLR = log-likelihood rate.

**Figure 6.**
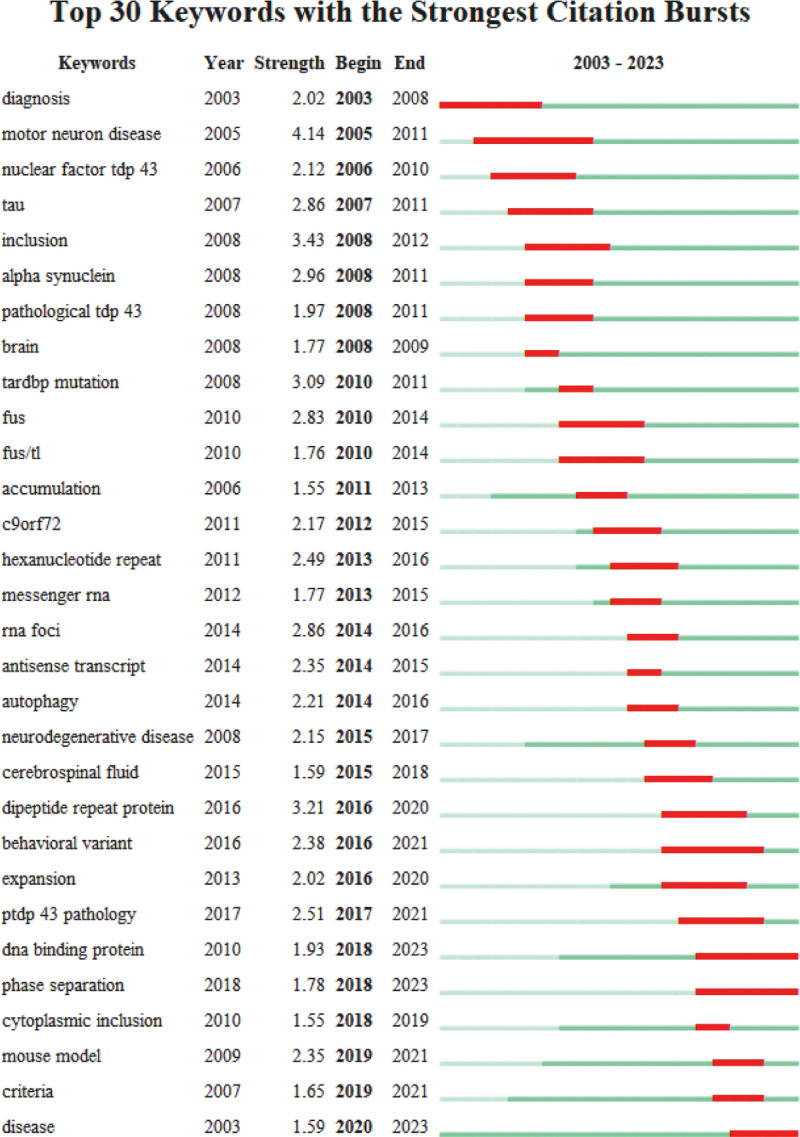
Top 30 keywords with the strongest citation explosion in FTLD-ALS literature. ALS = amyotrophic lateral sclerosis, FTLD = frontotemporal lobar degeneration.

“Keywords with the strongest citation bursts” usually refers to keywords or terms whose citation frequencies suddenly and significantly increase in a specific academic field or publication. These keywords indicate a sharp increase in the academic community’s attention and interest in a certain research field or topic. By analyzing the evolution of keywords (Fig. 6), we can find that “tdp-43” and “mutation” have always been research hotspots in ALS and FTLD. However, as time goes by, scholars have gradually shifted from the initial focus on clinical symptoms, disease diagnosis, and genetic research to more precise areas such as RNA, hippocampal sclerosis, and the C9orf72 gene. At the same time, the research on the phenomenon of phase separation has aroused widespread interest among scholars.

### 3.4. Top 10 citation publications

In order to accurately grasp the development trend, core research direction, and potential clinical application value in the field of FTLD-ALS, we analyzed the top 10 citation-ranked articles. Among these articles, 7 articles collectively focused on TDP-43, an important pathological protein in 2 neurodegenerative diseases, FTLD and ALS. The core findings of these 7 articles mainly focused on the multiple modifications (e.g., ubiquitination, phosphorylation), inclusion body formation of TDP-43 protein during the pathological process of FTLD and ALS, as well as the differences in its pathological features in different neurological regions.^[18–20,28–31]^ These studies revealed the key role of TDP-43 protein in the pathogenesis of FTLD-ALS, which provides an important basis for understanding the pathological basis of the disease, discovering diagnostic markers and exploring potential therapeutic targets. Other articles focus on the C9orf72 gene mutation and its related mechanism, revealing the important role of GGGGCC repeat amplification in C9orf72 gene in the pathogenesis of FTLD-ALS and the neurotoxicity mechanism of the resulting dipeptide-repeat proteins (DPRs).^[32–34]^ These studies further enriched the pathological spectrum of FTLD-ALS and provided new directions for the diagnosis and treatment of hereditary FTLD-ALS.

**Figure 7.**
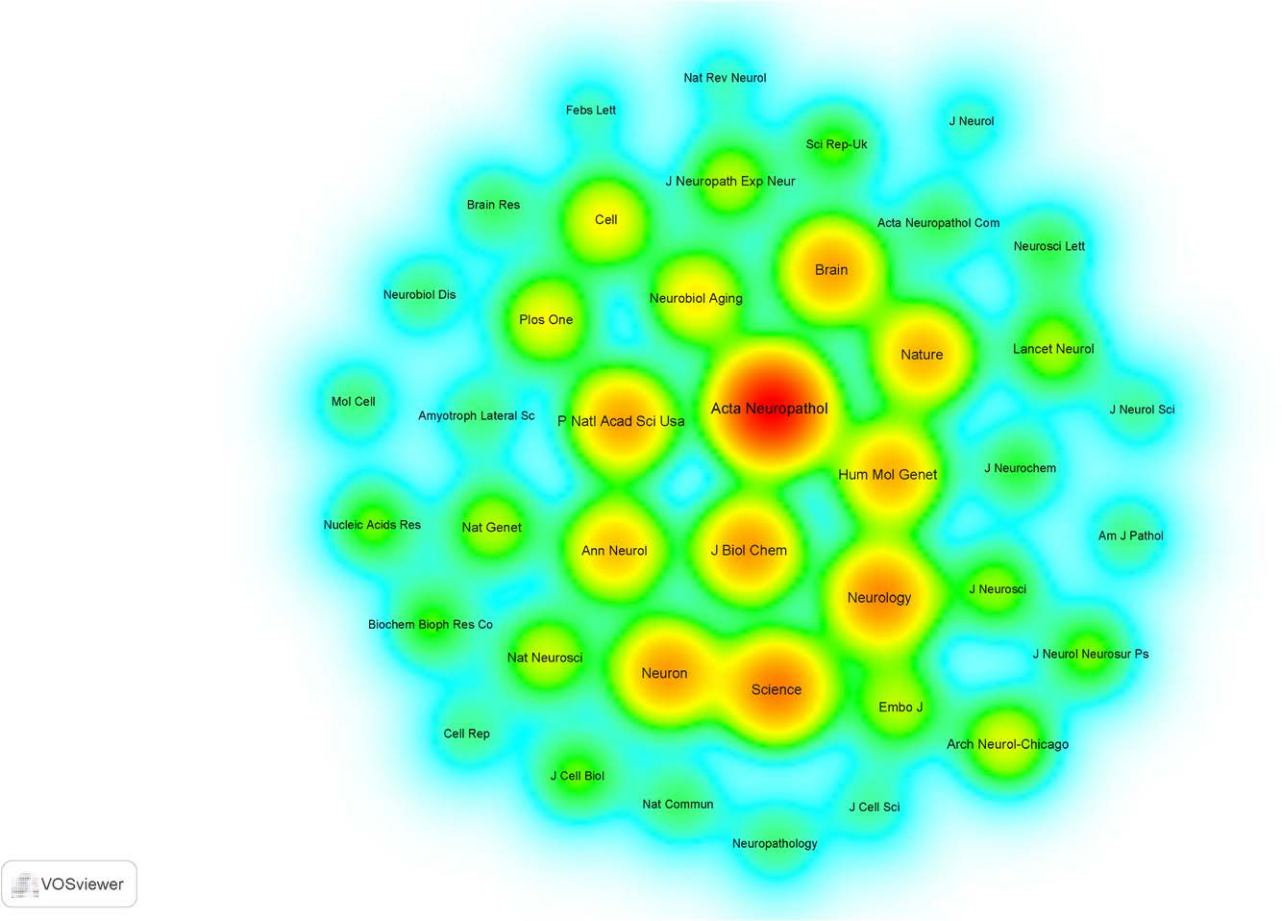
Mapping of highly co-cited journals in the FTLD-ALS field. ALS = amyotrophic lateral sclerosis, FTLD = frontotemporal lobar degeneration.

We further analyzed the co-cited references of 145 FTLD-ALS literatures to reveal the key research nodes and academic context in this field. Among the 5604 cited references, the minimum citation frequency for a single reference was set at 20 times, and 21 literatures reached this threshold (Table 2). The article “Ubiquitinated TDP-43 in frontotemporal lobar degeneration and ALS” written by Manuela Neumann was cited a total of 91 times. The study found that TDP-43 is the main disease protein in the 2 diseases, further demonstrating the crucial role of TDP-43 in these neurodegenerative diseases and providing important clues and a foundation for the research and treatment of related diseases.^[18]^ 16 Among the 855 journals from which the references were cited, the minimum citation frequency for a single journal was set at 50 times. By analyzing the number of citations of the journals, 41 journals reached the threshold (Fig. 7). Journals with the number of citations exceeding 300 times include Acta Neuropathol, Science, Neurology, J Biol Chem, Neuron, Brain, and P Natl Acad Sci USA, indicating that the literatures published in high-quality journals are more likely to be cited.

**Table 2:** Top 10 highly-cited literature in the FTLD-ALS field.

Rank	Titles	Authors	Journals	Number of citations (n)
1	Ubiquitinated TDP-43 in frontotemporal lobar degeneration and amyotrophic lateral sclerosis	Manuela Neumann	Science	91
2	TDP-43 is a component of ubiquitin-positive tau-negative inclusions in frontotemporal lobar degeneration and amyotrophic lateral sclerosis	Tetsuaki Arai	Biochem Biophys Res Commun	57
3	Expanded GGGGCC hexanucleotide repeat in noncoding region of C9ORF72 causes chromosome 9p-linked FTD and ALS	Mariely DeJesus-Hernandez	Neuron	45
4	TDP-43 mutations in familial and sporadic amyotrophic lateral sclerosis	Jemeen Sreedharan	Science	42
5	A hexanucleotide repeat expansion in C9ORF72 is the cause of chromosome 9p21-linked ALS-FTD	Alan E Renton	Neuron	39
6	Mutations in FUS, an RNA processing protein, cause familial amyotrophic lateral sclerosis type 6	Caroline Vance	Science	36
7	Mutations in the FUS/TLS gene on chromosome 16 cause familial amyotrophic lateral sclerosis	T J Kwiatkowski Jr	Science	34
8	Frontotemporal lobar degeneration: a consensus on clinical diagnostic criteria	D Neary	Neurology	32
9	TARDBP mutations in individuals with sporadic and familial amyotrophic lateral sclerosis	Edor Kabashi	Nat Genet	31
10	Converging mechanisms in ALS and FTD: disrupted RNA and protein homeostasis	Shuo-Chien Ling	Neuron	28

ALS = amyotrophic lateral sclerosis, FTD = frontotemporal dementia, FTLD = frontotemporal lobar degeneration, FUS = fused in sarcoma, RNA = ribonucleic acid, TARDBP = TAR DNA-binding protein, TDP-43 = TAR DNA-binding protein 43, TLS = translocated in liposarcoma.

## 4. Discussion

This study analyzed the development trend of FTLD-ALS related literatures. Firstly, in terms of the number of articles published, FTLD-ALS related research shows an increasing trend, especially reaching a peak in recent years. This indicates that researchers’ attention to these diseases is gradually increasing, and the research on their mechanisms and treatment methods is also deepening continuously. Secondly, from the perspective of countries and institutions, the United States, Japan, the United Kingdom, and Germany are the countries that have published the most relevant articles. The research institutions in these countries have strong strength and a research foundation in the FTLD-ALS field and have made significant contributions to the research of these diseases. It is particularly worth mentioning that the University of Pennsylvania and Ludwig Maximilian University of Munich are the institutions that have published the largest number of articles, demonstrating their leading position in this field. In addition, from the perspective of the citation frequency and cooperation network, Ludwig Maximilian University of Munich has the highest number of citations for the published FTLD-ALS related articles, and this institution has formed a large cooperation network with other institutions. This indicates that the research output of Ludwig Maximilian University of Munich in this field has been widely recognized and cited, and also demonstrates the importance of cross-institutional cooperation in promoting research progress. Finally, in terms of the distribution of research journals, Acta Neuropathologica is the journal that has published the largest number of FTLD-ALS related articles and has a high number of citations. Other journals such as Human Molecular Genetics, Neurobiology of Aging, and Neuropathology have also published related research. Researchers can choose appropriate channels for publishing their research achievements according to the academic influence and professionalism of these journals. Among the top 10 articles in terms of the number of citations, 7 articles focus on the role of TDP-43 in FTLD and ALS. The article with the highest number of citations points out that TDP-43 is the main pathological protein in FTLD and ALS and can be used together with ubiquitin antibodies to label various abnormal structures. This discovery reveals clues about the existence of a common pathological mechanism between FTLD and ALS.

Through the analysis of core authors, key words, and highly-cited literature, we believe that the current research in this field mainly focuses on the pathological mechanisms driven by TDP-43 proteinopathy, the complex pathogenic mechanisms of C9orf72 gene repeat expansion, RNA metabolism and nucleocytoplasmic transport disorders, autophagy-lysosome pathway defects, and the role of neuroinflammation and microglia/ astrocytes. For example, in terms of the pathological mechanisms driven by TDP-43 proteinopathy, the research has gradually shifted from the description of pathological phenomena to the in-depth analysis of molecular mechanisms (such as liquid-liquid phase separation (LLPS) regulation and nucleocytoplasmic transport disorder) and the development of targeted therapeutic strategies (such as small-molecule-mediated inhibition of aggregation and gene-editing-mediated restoration of TDP-43 function), and single-cell sequencing and organoid models are used to explore pathological spread and cell-type-specific toxicity. Regarding the complex pathogenic mechanisms of C9orf72 gene repeat expansion, scholars currently focus on its triple-toxicity mechanism (RNA foci formation, dipeptide-repeat protein deposition, and C9orf72 haploinsufficiency), revealing that it drives disease progression through nucleocytoplasmic transport disorders, autophagy-lysosome function inhibition, and neuroinflammatory cascades. They are also actively exploring clinical translation paths such as antisense oligonucleotide-mediated targeted silencing of repeat RNA, CRISPR/ Cas9-mediated gene-editing-mediated cleavage of expanded sequences, and immunotherapy-mediated neutralization of toxic DPR proteins. Within the scope of research on RNA metabolism and nucleocytoplasmic transport disorders, researchers have deeply analyzed the abnormal phase separation of RNA-binding proteins (typically FUS and TDP-43) and the functional defects of nuclear transport receptors (i.e., transportin/importin). Studies have shown that these abnormal conditions, through the LLPS mechanism, promote the abnormal solidification of RNA granules and damage the nuclear pore complex, ultimately interfering with the splicing, localization, and translation homeostasis of mRNA. To further explore the differences in the sensitivity of neuronal subsets to RNA metabolic defects, researchers use single-cell multi-omics technology in combination with iPSC-organoid models for research. On this basis, a series of targeted intervention strategies have been developed, including the use of antisense oligonucleotides (ASO) to correct abnormal mRNA splicing, the use of small molecules to regulate the LLPS dynamic process (such as inhibiting the aggregation of the TDP-43-C-terminal fragment), and the use of gene-editing technology to target nuclear localization signals for repair. Although the current research has made important breakthroughs at the molecular level in analyzing the core pathological mechanisms such as TDP-43 and C9orf72, there are still many problems to be solved, such as the challenges of disease heterogeneity, the interaction of multiple systems, and the obstacles to clinical translation. Through the combination of keyword analysis, we believe that the research in the FTLD-ALS field will transform and develop towards a multi-dimensional and cross-scale systematic research paradigm. It will specifically focus on the following directions: the spatiotemporal dynamic analysis of pathological mechanisms, the study of disease heterogeneity and precise typing, the research on multi-system interaction networks, the breakthrough of clinical translation technologies, and the innovation driven by computational medicine and AI, ultimately achieving a paradigm innovation from “single-target-universal-therapy” to “mechanism-network-precise-intervention.”

In terms of data sources, using only the Web of Science database is rather limited, and it may lead to the omission of relevant research findings in other databases. Secondly, using the number of citations to measure the importance of research also has limitations. Newly published research usually has fewer citations due to the short time since publication, while earlier research may be over-estimated because of its first-mover advantage. In addition, when different researchers conduct keyword co-occurrence analysis and cluster analysis, there may be biases in the interpretation of indicators, which affects the accuracy of research results. However, our research has been carefully planned and carried out, and compared with the existing research in the relevant field to ensure that our results are of high accuracy and reliability. In the process of literature screening, keyword co-occurrence analysis and cluster analysis, we have carried out multiple rounds of discussion and review to minimize the bias caused by subjective factors of different personnel. Overall, we believe that our research results are highly reliable and valuable, providing meaningful references and inspirations for the research in the relevant field. In the future, when scholars conduct relevant research, they can draw on our research methods and experience, further expand the data sources, improve the importance measurement criteria, and reduce the subjective bias, so as to promote the continuous progress of the research in this field.

## 5. Conclusion

To our knowledge, this is the first econometric evaluation of the literature on FTLD-ALS. Current studies in this field have focused on the pathogenesis driven by TDP-43 proteopathy, the complex pathogenesis of C9orf72 gene repeat amplification, RNA metabolism and impaired nucleoplasmic transport, defects in autophagy-lysosome pathway, and neuroinflammation and microglia/astrocyte roles. These studies have provided important clues and foundations for our in-depth understanding of the pathogenesis as well as therapeutic approaches of FTLD-ALS. In the future, we will further focus on the analysis of spatial and temporal dynamics of FTLD-ALS pathogenesis, heterogeneity and precision typing of FTLD-ALS, multi-system interaction network research, breakthroughs in clinical translational technology, and computational medicine and AI-driven innovations, etc, so as to ultimately realize the transition from “single target-universal therapy” to “mechanism-network-precision intervention” and to “mechanism-network-precision intervention.” Ultimately, we will realize the paradigm innovation from “single target-universal therapy” to “mechanism network-precise intervention.”

## Acknowledgments

We acknowledge the use of software tools such as Microsoft Excel, CiteSpace, VOSviewer, and Scimago Graphica in our research.

## Author contributions

**Conceptualization:** Gaihong Liu, Ningning Liu.

**Data curation:** Gaihong Liu, Ningning Liu, Yingxue Xu.

**Formal analysis:** Gaihong Liu, Ningning Liu, Yingxue Xu.

**Funding acquisition:** Gaihong Liu, Ningning Liu, Yingxue Xu.

**Investigation:** Gaihong Liu, Ningning Liu, Yingxue Xu.

**Methodology:** Gaihong Liu, Ningning Liu.

**Project administration:** Yingxue Xu.

**Supervision:** Fan Su.

**Validation:** Fan Su.

**Visualization:** Fan Su.

**Writing – original draft:** Gaihong Liu, Yingxue Xu.

**Writing – review & editing:** Fan Su.
